# From Active to Non-active Giant Cell Arteritis: Longitudinal Monitoring of Patients on Glucocorticoid Therapy in Combination With Leflunomide

**DOI:** 10.3389/fmed.2021.827095

**Published:** 2022-01-20

**Authors:** Tadeja Kuret, Mojca Frank-Bertoncelj, Katja Lakota, Polona Žigon, Gerhard G. Thallinger, Andreja N. Kopitar, Saša Čučnik, Matija Tomšič, Alojzija Hočevar, Snežna Sodin-Šemrl

**Affiliations:** ^1^Institute of Cell Biology, Faculty of Medicine, University of Ljubljana, Ljubljana, Slovenia; ^2^Department of Rheumatology, University Medical Centre Ljubljana, Ljubljana, Slovenia; ^3^Faculty of Mathematics, Natural Science and Information Technologies, University of Primorska, Koper, Slovenia; ^4^BioMed X Institute, Heidelberg, Germany; ^5^Institute for Biomedical Informatics, Graz University of Technology, Graz, Austria; ^6^OMICS Center Graz, BioTechMed Graz, Graz, Austria; ^7^Institute of Microbiology and Immunology, Faculty of Medicine, University of Ljubljana, Ljubljana, Slovenia; ^8^Faculty of Pharmacy, University of Ljubljana, Ljubljana, Slovenia

**Keywords:** giant cell arteritis, glucocorticoids, leflunomide, follow-up, biomarkers, disease monitoring

## Abstract

In the present study, we longitudinally monitored leukocyte subsets, expression of neutrophil surface adhesion molecules (CD62L and CD11b) and serum analytes in therapy-naïve patients with active giant cell arteritis (GCA). We collected blood samples at the baseline, and at weeks 1, 4, 12, 24, and 48 of follow-up, and evaluated short- and long-term effects of glucocorticoids (GC) vs. GC and leflunomide. Our aim was to identify candidate biomarkers that could be used to monitor disease activity and predict an increased risk of a relapse. Following high doses of GC, the numbers of CD4+ T-lymphocytes and B-lymphocytes transiently increased and then subsided when GC dose tapering started at week 4. In contrast, the numbers of neutrophils significantly increased during the follow-up time of 12 weeks compared to pre-treatment time. Neutrophil CD62L rapidly diminished after initiation of GC therapy, however its expression remained low at week 48, only in patients under combinatorial therapy with leflunomide. Levels of acute phase reactant SAA and IL-6 decreased significantly after treatment with GC and leflunomide, while levels of IL-8, IL-18, and CHI3L1 did not change significantly during the follow-up period. CHI3L1 was associated with signs of transmural inflammation and vessel occlusion and might therefore serve as a marker of fully developed active GCA, and a promising therapeutic target. Patients with relapses had higher levels of IL-23 at presentation than patients without relapses (*p* = 0.021). Additionally, the levels of IL-23 were higher at the time of relapse compared to the last follow-up point before relapse. IL-23 might present a promising biomarker of uncontrolled and active disease and could give early indication of upcoming relapses.

## Introduction

Giant cell arteritis (GCA) is a granulomatous vasculitis affecting large- and medium-sized arteries (1). In the majority of patients, cranial and extracranial large arteries are involved to different degrees, leading to specific clinical phenotypes (2). Predominant cranial GCA (C-GCA) is characterized by headache, jaw claudication and visual disturbances, while clinical signs and symptoms of extra-cranial [large vessel (LV-GCA)] typically include weight loss, myalgia and fever (3). Erythrocyte sedimentation rate (ESR) and/or levels of C-reactive protein (CRP) are usually increased at presentation in GCA patients, indicating a strong acute inflammatory response (4, 5).

High dose glucocorticoids (GC) represent the first-line treatment for GCA (6). They effectively control systemic inflammation and successfully prevent ischemic complications, such as acute vision loss. Relapses, however, are common when GC tapering regimen is applied, most likely due to ongoing inflammation in the affected vascular tissues, not adequately suppressed by GC (7). In addition, long-term use of GC is associated with various adverse effects, including bone fractures, infections, diabetes mellitus, and hypertension (8). Several other disease modifying anti-rheumatic drugs have subsequently been investigated for their steroid-sparing effect in GCA (9). So far, only tocilizumab showed the efficacy for achieving a sustained remission at week 52 of follow-up compared to placebo (10, 11). However, it was recently discovered by magnetic resonance angiography that signs of vascular inflammation persist in two-thirds of GCA patients treated with tocilizumab, despite clinical remission (12). Other agents, such as methotrexate exhibited limited or no evidence of benefit in the treatment of GCA (13). Leflunomide, on the other hand, has been shown to be effective and safe in reducing the rate of relapses in GCA in a small open-label study (14).

Classical acute phase parameters, such as ESR and CRP are commonly used for monitoring GCA activity (15, 16). However, measuring ESR and CRP to predict relapses has a limited value, since GC strongly suppress the systemic acute phase response, decrease ESR, as well as serum levels of CRP, despite an ongoing local vascular inflammation (17, 18). Subsequently, one cannot determine whether patients in GC-free remission are truly in remission or are still suffering from an ongoing subclinical disease. Therefore, new biomarkers are needed to predict the risk of relapses, and monitor disease activity in patients with GCA.

Van Sleen et al. (19) demonstrated higher numbers of monocytes and neutrophils, and lower numbers of natural killer (NK) and B cells in therapy-naïve GCA patients compared to healthy blood donors (HBDs). During GC treatment, as well as in GC-free remission, myeloid subsets remained elevated, while lymphoid subsets fluctuated substantially (19). Additionally, an altered phenotype of circulating neutrophils was also reported. The neutrophil phenotype changed from activated and highly adhesive, in the early stages of GCA, to a less adhesive after 48 h of GC treatment. However, 24 weeks following GC treatment and therapy tapering, neutrophils with the activated phenotype reappeared exhibiting a high expression of adhesion molecules L-selectin (CD62L) and integrin αM (CD11b) (20). Long-term monitoring of the neutrophil phenotype could point to an incompletely controlled disease process (e.g., relapse) (21).

Previously, our cross-sectional study revealed significantly higher levels of serum amyloid A (SAA), interleukin (IL)-6, IL-8, IL-18, IL-23 and chitinase 3 like protein 1 (CHI3L1) in sera of therapy-naïve GCA patients compared to HBDs, reflecting an active disease (22). SAA has recently gained more attention in GCA (23), since it has been found to be highly elevated in GCA patients with active vs. inactive disease (24), and associated with relapses and visual disturbances (22).

In the current study, we longitudinally monitored the quantitative changes in leukocyte subtypes, neutrophil expression of adhesion molecules (CD62L, CD11b) and serum levels of selected analytes in GCA patients to evaluate the short- and long-term effects of GC vs. GC and leflunomide. Our aim was to identify candidate cellular and molecular biomarkers that could help monitoring disease activity and predicting the risk of a relapse.

## Methods

### Patients

**Figure 1:**
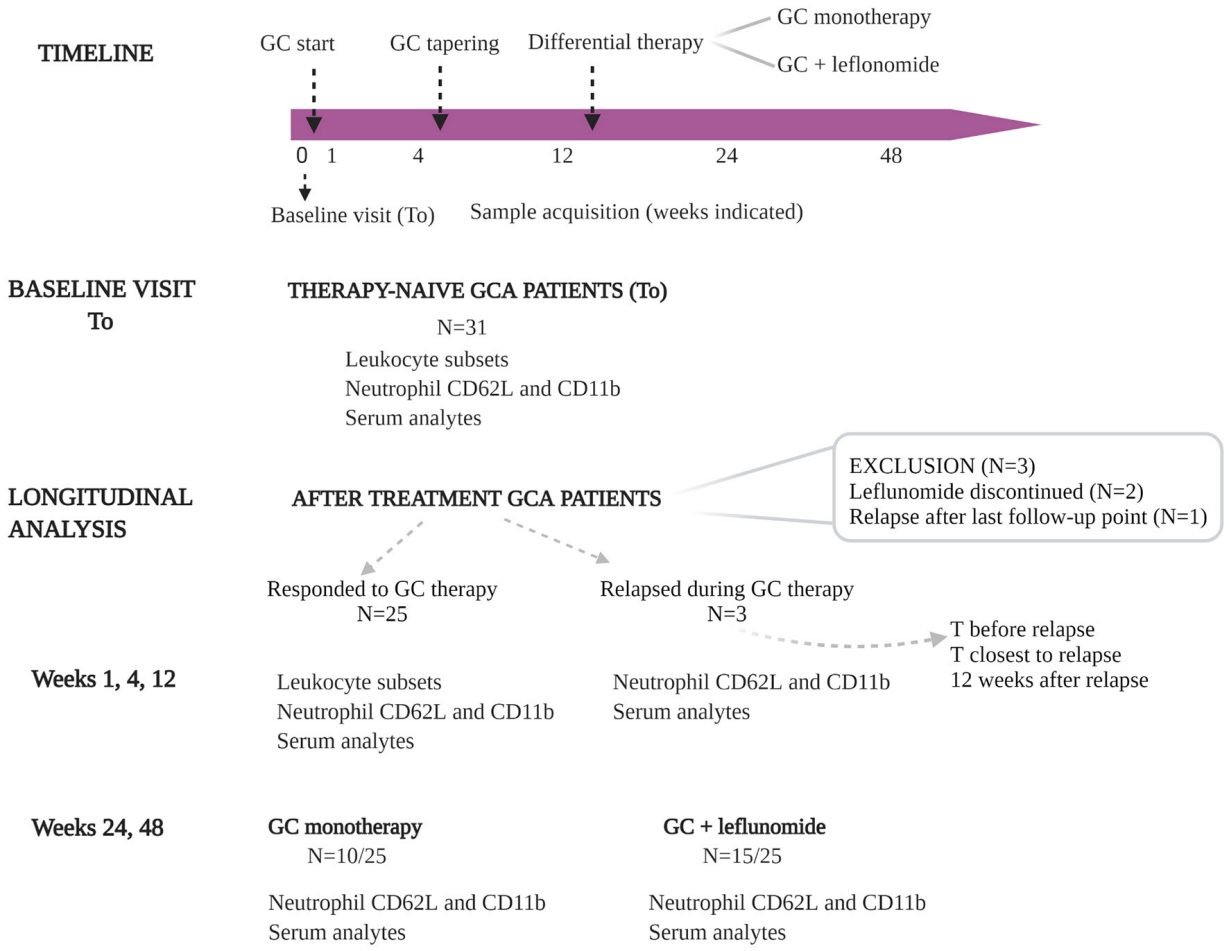
Overview of the study design. Thirty-one consecutive, therapy-naïve GCA patients were included in the study. Blood samples from GCA patients were obtained at baseline visit [before initiation of GC therapy (T_0_)], and during follow-up at weeks 1, 4, 12, 24, and 48. GC treatment was initiated at the time of diagnosis. GC tapering started at week 4 after sampling. Leflunomide (10 mg/day) was introduced as an adjuvant therapy at week 12 to 17 GCA patients (two of them experienced therapy-related adverse events and were excluded from longitudinal analysis). During follow-up and GC tapering, three of 25 patients experienced disease relapse after they already responded to GC therapy. At the time of relapse these patients were on GC monotherapy and consequently received leflunomide (10 mg/day), in addition to GC. From 3 relapsing patients, the data was collected before relapse, at the time closest to relapse and 12 weeks after relapse. GC, glucocorticoids; GCA, giant cell arteritis; T, time.

Thirty-one consecutive therapy-naïve GCA patients were enrolled in the study between October 2016 and October 2017. The diagnosis was established based on the 1990 ACR classification criteria (25) and a positive temporal artery biopsy (TAB) or positive color Doppler sonography (CDS) of temporal arteries. Blood samples from GCA patients were obtained at baseline visit [before initiation of GC therapy (T_0_)], as well as during follow-up at weeks 1, 4, 12, 24 and 48, unless otherwise stated. GC treatment was initiated at the time of diagnosis (Figure 1) in accordance with the unified protocol following the EULAR guidelines (26). Tapering of GC started at week 4 after baseline visit. Leflunomide (10 mg/day) was introduced as an adjuvant therapy at the week 12 to 17/31 patients. 2/17 patients experiencing adverse events (e.g., hair loss, diarrhea), discontinued leflunomide therapy and were consequently excluded from the longitudinal analysis. During follow-up and GC tapering, 4/31 patients experienced disease relapse after having already responded to GC therapy. Relapse was defined as the need for treatment intensification following new or increasing clinical symptoms typical of GCA. At the time of relapse, these patients were on GC monotherapy and consequently received leflunomide (10 mg/day), in addition to GC. From 3 relapsing patients, data was collected at the time before relapse (in remission), at the time closest to relapse (active disease) and 12 weeks after relapse (in remission). One patient relapsed in week 57 after diagnosis (after the last study follow-up point) and was excluded from the longitudinal analysis due to missing data (Figure 1). Patients and their samples were anonymized, before being used in the analyses. All patients signed informed consent to participate in the study. The study was approved by the Slovenian National Medical Ethics Committee (#99/04/15 and #65/01/17).

### Histological Examination of Temporal Artery Biopsies and Routine Laboratory Parameters

Histological analyses were performed on formalin-fixed paraffin-embedded sections stained with hematoxylin and eosin. Arterial wall inflammatory infiltrate and arterial occlusion were semiquantitatively scored. Arterial occlusion was considered when luminal stenosis was >75%.

Among laboratory parameters, ESR was measured by the WesternGreen method, CRP using Siemens Advia colorimetric assay, fibrinogen was detected by Siemens BCS XP/modified Clauss method, ferritin using the Advia chemiluminescence assay, SAA and haptoglobin were determined using immunonephelometry from Siemens (BN Prospec System and BN II, respectively).

### Flow Cytometry

Venous blood was drawn from GCA patients into heparin-containing tubes. Whole blood immunophenotyping was performed using 7-Color Immunophenotyping kit with the following antibodies (Miltenyi Biotec, catalog #130-098-456): CD14-FITC (clone Tük4), CD56-PE (clone REA196), CD16-PE (clone REA423), CD4-PerCP (clone VIT4), CD19-PE-Vio® 770 (clone LT19), CD3-APC (clone BW264/56), CD8-APC-Vio 770 (clone BW135/80), CD45-VioBlue® (clone 5B1). Briefly, 100 μl of whole blood was incubated with 10 μl immunophenotyping reagent for 10 min in the dark, at 4°C. After incubation, whole blood was lysed using Red Blood Lysing Solution (Miltenyi Biotec, catalog #130-098-456). Neutrophil phenotyping was performed in 50 μl of whole blood, incubated for 30 min at 4°C in the dark, with the following antibodies (eBioscience): CD16-PE (clone eBioCB16; catalog #50-112-4738), CD62L-PE-Cy5 (clone DREG56; catalog #50-140-71) and CD11b-APC (clone ICRF44; catalog #17-0118-42). After incubation, samples were lysed, using Whole Blood Lysing Reagent Kit (Beckman Coulter; catalog #6602764). All samples were analyzed using flow cytometer MACSQuant Analyzer 10 (Miltenyi Biotec). Analysis of flow cytometry data was performed using MACSQuantify (Analysis Software version 2.8, Miltenyi Biotec) and FlowLogic (Flow Cytometry Analysis Package, version 7.00.0a, Invasion Software Technologies Pvt Ltd).

### Biomarker Protein Detection

Serum concentrations of IL-8, IL-18, IL-23, CHI3L1 and soluble CD62L (sCD62L) were measured by MagPix (Luminex xMAP Technology) using human pre-mixed multi-analyte kits (R&D Systems; catalog #LXSAHM) and IL-6 using ELISA (Invitrogen; catalog #KHC0061).

### Statistical Analysis

Statistical analysis was performed using SPSS statistical software package version 22.0 and Graph Pad Prism software 9.0. The normality of data distribution was investigated by the Shapiro-Wilk test. Due to the non-normal distribution of the data, summary statistics are expressed as medians and 25–75th percentiles (Q_25_-Q_75_). Mann-Whitney U-test was used to compare medians of measured parameters in GCA patients with or without specific clinical signs/symptoms. Statistical analysis of longitudinal data was performed using Kruskal-Wallis test followed by Dunn's multiple comparison test, which calculates adjusted *p*-values. All tests were two-tailed and *p*-values of <0.05 were regarded as statistically significant.

## Results

### Baseline Visit

The median (Q_25_-Q_75_) age of the included patients was 74.9 (68.0–76.8) and there were 20 (65%) females. The most frequent clinical symptoms/signs reported were newly formed headache (74%), jaw claudication (65%) and general symptoms (71%). Visual disturbances were present in eight (26%) patients and seven patients (23%) had LV-GCA. The median (Q_25_-Q_75_) ESR was 78.0 (48.0–94.5) mm/h and the median CRP value was 71.5 (34.3–128.8) mg/l (Table 1).

**Table 1:** Demographics, clinical and laboratory data of therapy-naïve GCA patients at baseline visit.

**Demographic data**	
Number of patients	31
Median age in years (Q_25_-Q_75_)	74.9 (68.0–76.8)
Number of females (%)	20 (65)
Median duration of symptoms (days) (Q_25_-Q_75_)	30 (30–60)
Median body mass index (kg/m^2^) (Q_25_-Q_75_)	23.8 (21.3–28.7)
**Symptoms and signs *n* (%)**	
General symptoms	22 (71)
Fever	5 (16)
Weight loss	19 (61)
Headache	23 (74)
Jaw claudication	20 (65)
Scalp tenderness	12 (39)
Visual disturbances	8 (26)
Dry cough	5 (16)
Large vessel involvement	7 (23)
**Histological examination of TABs *n* (%)**	
TAB performed	23 (74)
Transmural inflammation	18 (78)
Vessel occlusion	8 (34)
**Ultrasound examination of temporal arteries *n* (%)**	
HALO effect	28 (90)
**Median laboratory values (Q_25_-Q_75_)**	
ESR (mm/h)	78.0 (48.0–94.5)
CRP (mg/l)	71.5 (34.3–128.8)
Fibrinogen (g/l)	6.2 (5.7–7.2)
Ferritin (g/l)	258 (161–441)
Haptoglobin (g/l)	4.5 (2.6–5.6)

*CRP, C-reactive protein; ESR, erythrocyte sedimentation rate; GCA, giant cell arteritis; TAB, temporal artery biopsy*.

#### Therapy-Naïve GCA Patients With Transmural Inflammation and Occlusion of Temporal Arteries Have Higher Serum Levels of CHI3L1

To reveal if the inflammatory process in TABs of therapy-naïve GCA patients associates with the numbers of leukocyte subsets and serum parameters, we correlated the measured baseline cell and serum parameters with the presence of histological signs of GCA (transmural inflammation, occlusion of temporal arteries), clinical symptoms and signs and development of a future relapse.

**Figure 2:**
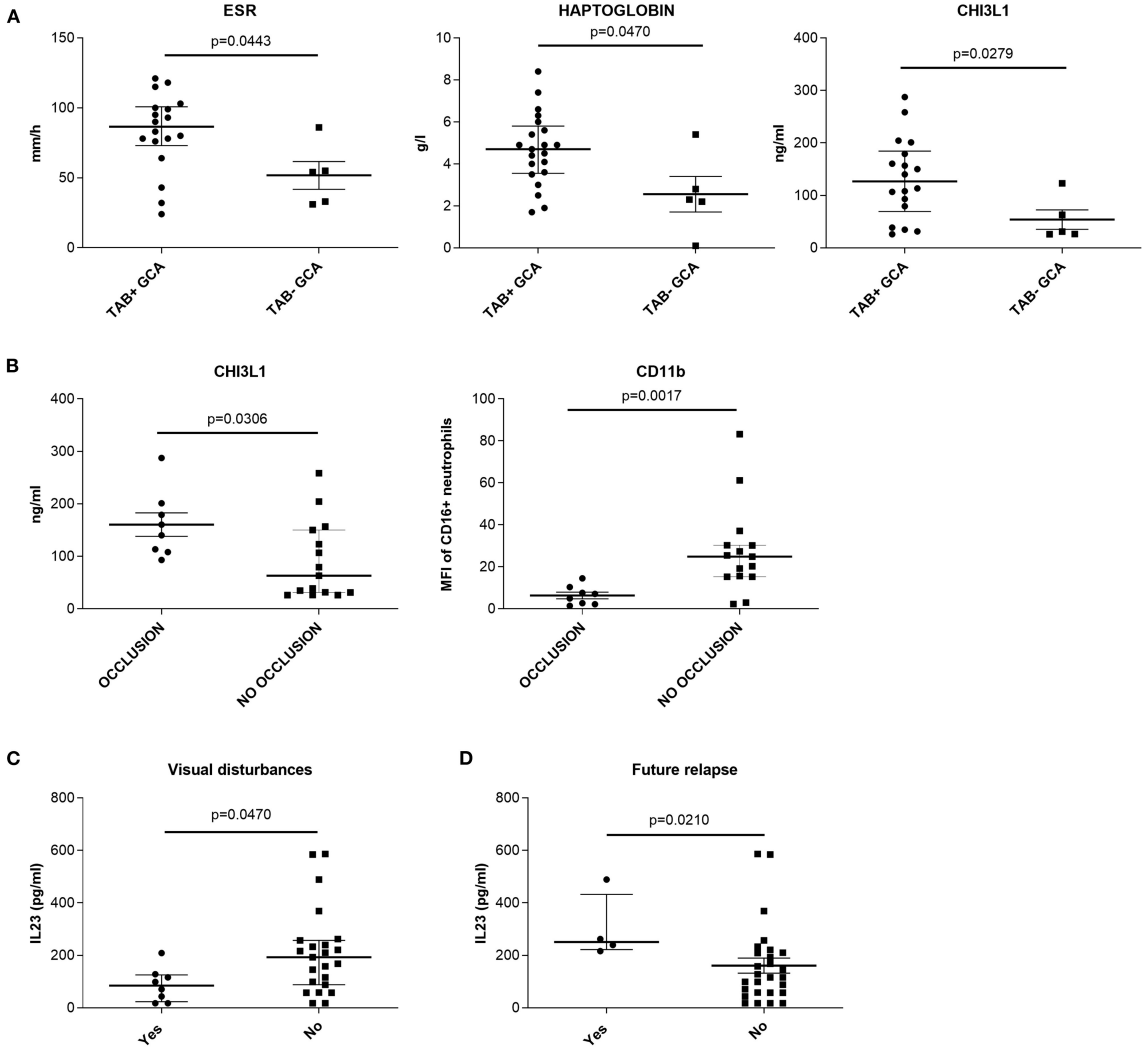
Association between histopathological findings in temporal artery biopsies of therapy-naïve GCA patients and **(A,B)** presence of visual disturbances; **(C)** development of a future relapse and **(D)** measured cell and serum parameters. **(A)** medians, Q_25_ and Q_75_ in patients with (TAB + GCA) or without (TAB− GCA) signs of transmural inflammation in the temporal arteries. **(B)** medians, Q_25_ and Q_75_ in patients with or without occlusion of the temporal arteries. **(C)** medians, Q_25_ and Q_75_ in patients with or without visual disturbances. **(D)** medians, Q_25_ and Q_75_ in patients with or without future relapse. CHI3L1, chitinase 3 like protein 1; ESR, erythrocyte sedimentation rate; GCA, giant cell arteritis; IL, interleukin; MFI, median fluorescence intensity; TAB, temporal artery biopsy.

Histological examination of TAB was performed in 23 therapy-naïve GCA patients. Signs of transmural inflammation (TAB + GCA) and vessel occlusion were found in 18 (78%) and 8 (26%) of the examined TABs, respectively (Table 1). In general, the patients with transmural inflammation had significantly higher ESR (*p* = 0.0443), haptoglobin (*p* = 0.0470) and CHI3L1 (*p* = 0.0279) compared to patients with no signs of inflammation in the TABs (TAB- GCA) (Figure 2A). GCA patients with occlusion of the temporal arteries had higher amount of CHI3L1 (*p* = 0.0306) but lower neutrophil expression of CD11b (*p* = 0.0017) compared to GCA patients without vessel occlusion (Figure 2B). Other measured parameters (serum analytes, the number of leukocyte subsets, the expression of CD62L) did not associate with temporal artery transmural inflammation or occlusion.

Correlating the clinical signs and symptoms with the measured parameters, we found that therapy-naïve GCA patients with visual disturbances (*n* = 8) had lower median amount of serum IL-23 compared to patients without visual disturbances (*n* = 23; *p* = 0.047, Figure 2C). Additionally, the median amount of IL-23 was significantly higher in the serum of therapy-naïve GCA patients (at baseline visit) who developed a future relapse (*n* = 4) compared to patients with no future relapses (*n* = 27; *p* = 0.021) (Figure 2D). Other clinical symptoms and signs at baseline visit did not correlate with measured cell and serum parameters.

### Longitudinal Follow-Up

The longitudinal analysis included 28 of the initial 31 GCA patients, who received GC immediately after pre-treatment (baseline) sampling. Among them, 25 responded to GC at the initial dosage, with prompt improvement of clinical signs and symptoms of GCA, as determined by a rheumatologist during the follow-up visits. These patients were able to continue GC reduction without any deviation from the protocol. Three patients (9.7%) experienced a relapse following a period of remission during the 48 weeks of follow-up. Patient 1 (P1) experienced a relapse at week 24, P2 relapsed at week 48 and P3 relapsed at week 12. All relapsing patients were on GC monotherapy at the time of relapse. P1 and P2 received GC dosage of 4 mg/day, while P3 received 12 mg/day. All three relapses were characterized by new or intensified clinical symptoms considered typical of GCA. P1 and P2 experienced signs of systemic inflammation (fever, weight loss, fatigue, myalgia), while P3 experienced cranial signs (headache, jaw claudication). P1 and P2, but not P3, also had increased ESR and CRP, associated with GCA in the absence of an alternative explanation, compared to the last time point before relapse when they were in remission. All relapsing patients responded to additional therapy with leflunomide (10 mg/day), and were able to continue with GC tapering as scheduled.

#### Longitudinal Analysis Shows Fluctuation in Leukocyte Subsets From Active (Pre-treatment) to Non-active (After Treatment) GCA

**Figure 3:**
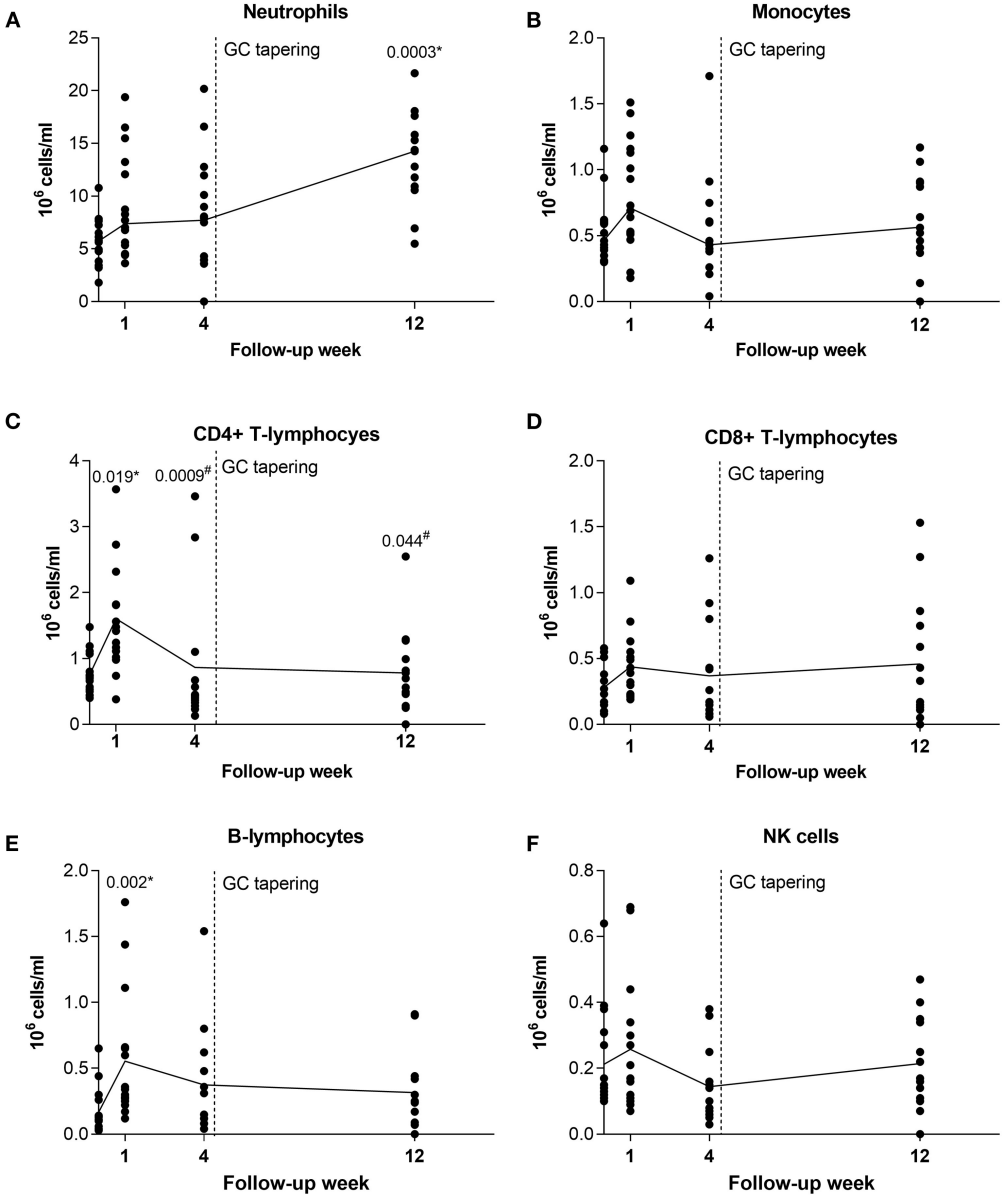
Longitudinal analysis of leukocyte subsets in GCA patients before and during therapy with GC. After pre-treatment sampling, all patients received GC and therapy tapering started after week 4 (indicated with vertical dotted line). Shown are medians from each follow-up time point (black horizontal connecting line) for **(A)** neutrophils; **(B)** monocytes; **(C)** CD4 + T-lymphocytes; **(D)** CD8+ T-lymphocytes; **(E)** B-lymphocytes; and **(F)** NK cells. GC, glucocorticoids; GCA, giant cell arteritis; NK, natural killer. * indicates statistical significance between the corresponding timepoint and T_0_; ^#^ indicates statistical significance between the corresponding timepoint and week 1.

To get insight into the effects of GC treatment on alterations of leukocyte subset composition during GC therapy, we obtained longitudinal profiling data for immune cells at T_0_ and 1, 4, and 12 weeks of follow up for 16 GCA patients. These patients were on GC monotherapy and responded to GC treatment. At week 1 after GC treatment, the number of circulating CD4+ T-lymphocytes (*p* = 0.019) and B-lymphocytes (*p* = 0.002) significantly increased in GCA patients compared to T_0_. The number of CD4+ T-lymphocytes then diminished, reaching the pre-treatment (T_0_) numbers at weeks 4 (*p* = 0.0009 vs. week 1) and 12 (*p* = 0.044 vs. week 1), while the number of B-lymphocytes only slightly decreased. In contrast, the number of neutrophils progressively increased over the weeks 1 and 4 and was significantly elevated at week 12 compared to T_0_ (*p* = 0.0003). No significant differences were observed in the number of monocytes, CD8+ T-lymphocytes and NK cells during the 12 weeks of follow-up (Figure 3).

#### Neutrophil Adhesion Molecules Are Differentially Expressed in Active Compared to Non-active GCA

To confirm the effect of GC and leflunomide on neutrophil phenotype, we measured neutrophil CD62L and CD11b expression in peripheral blood of 25 GCA patients who responded to therapy, at T_0_ and at weeks 1, 4, 12, 24, and 48 of follow-up.

**Figure 4:**
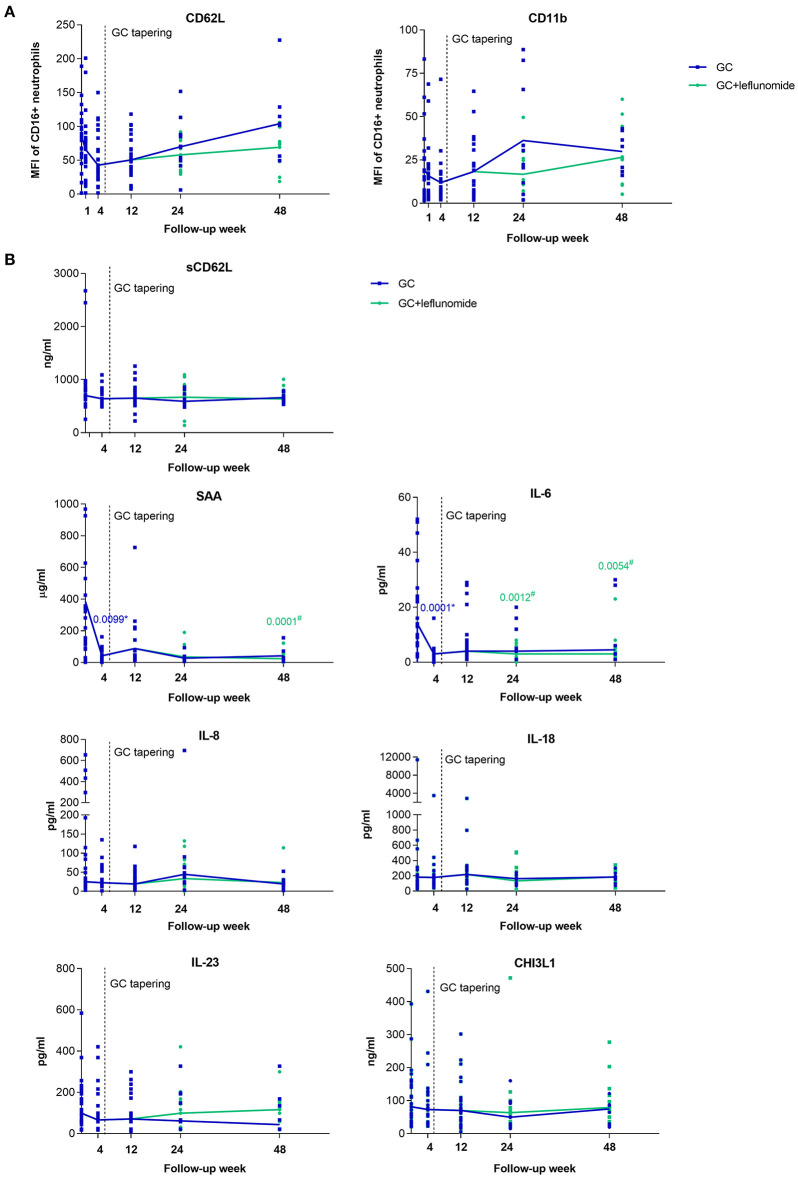
Surface expression of CD62L and CD11b on neutrophils **(A)** and serum levels of selected analytes **(B)** from GCA patients who responded to therapy (*n* = 25), at baseline visit (To) and 1, 4, 12, 24, and 48 weeks of follow-up. After pre-treatment sampling (time point 0), all patients received GC and therapy tapering started after week 4 (indicated with vertical dotted line). After week 12 some of the patients (15/25) received leflunomide, in addition to GC therapy (green). The horizontal lines connect the medians from each follow-up time point. GCA, giant cell arteritis; MFI; median fluorescence intensity. * indicates statistical significance between the corresponding timepoint and T_0_ in patients receiving GC monotherapy (blue); ^#^ indicates statistical significance between the corresponding timepoint and T_0_ in patients receiving GC in combination with leflunomide (green).

No significant differences in the neutrophil CD62L and CD11b expression were observed at T_0_ or at week 12, prior to leflunomide addition, between patients receiving GC and patients who later received GC and leflunomide (Supplementary Table 1). The median expression of CD62L on neutrophils, decreased in GCA patients, responding to therapy, from T_0_ to weeks 1, 4, and 12 after GC treatment (Figure 4A). At week 48, there was a distinct elevation in CD62L: patients receiving GC only, showed a marked increase, compared to T_0_, while in patients additionally receiving leflunomide, the CD62L expression was consistently low. However, the difference between the two groups was not significant. The median neutrophil expression of CD11b declined from T_0_ to week 4, but increased and reached median pre-treatment expression levels at week 12. Patients receiving GC only, showed an increase in CD11b expression at week 24 compared to patients receiving leflunomide in addition to GC, however both treatment groups exhibited similar CD11b expression at week 48 (Figure 4A). The differences in CD11b expression between different time points or differential therapy groups were not statistically significant.

Since the activation of neutrophils causes CD62L shedding from the membrane into the bloodstream (23), we also measured serum levels of sCD62L at baseline and during the follow-up of GCA patients. In contrast to the changing expression of neutrophil CD62L, median levels of sCD62L remained constant during the entire follow-up time (Figure 4B). No correlation between neutrophil CD62L and sCD62L was determined at T_0_ or any of the follow-up points (Supplementary Figure 1).

#### Serum Biomarker Levels Decrease From Active to Non-active GCA Depending on the Type of Treatment

To identify serum biomarkers that can be used to monitor GCA activity and could inform on the ongoing vascular inflammation, we determined serum levels of a predefined set of proteins (SAA, IL-6, IL-8, IL-18, IL-23, CHI3L1) in 25 GCA patients who responded to therapy at T_0_ and at weeks 4, 12, 24, and 48 of follow-up.

No significant differences in measured analytes were observed at T_0_ or at week 12, prior to leflunomide addition, between patients receiving GC and patients who later received GC and leflunomide (Supplementary Table 1). Importantly, the median levels of SAA (*p* = 0.0099) and IL-6 (*p* = 0.0001) decreased significantly at week 4 after GC treatment compared to T_0_. Both analytes remained low at weeks 12, 24, and 48 compared to T_0_ in both groups of patients although significance was only reached for patients under combinatorial therapy with leflunomide (*p* = 0.0001 for SAA at week 48; *p* = 0.0012 and *p* = 0.0054 for IL-6 at weeks 24 and 48, respectively). Median levels of serum IL-8, IL-18 and CHI3L1 remained stable during the entire follow-up, regardless of therapy used. Median IL-23 decreased from T_0_ to week 48 in patients receiving GC monotherapy, while in patients under combinatorial therapy there was an increase at weeks 24 and 48 compared to week 12, although this was not significant (Figure 4B).

#### IL-23 Is Increased in Relapsing Patients

**Figure 5:**
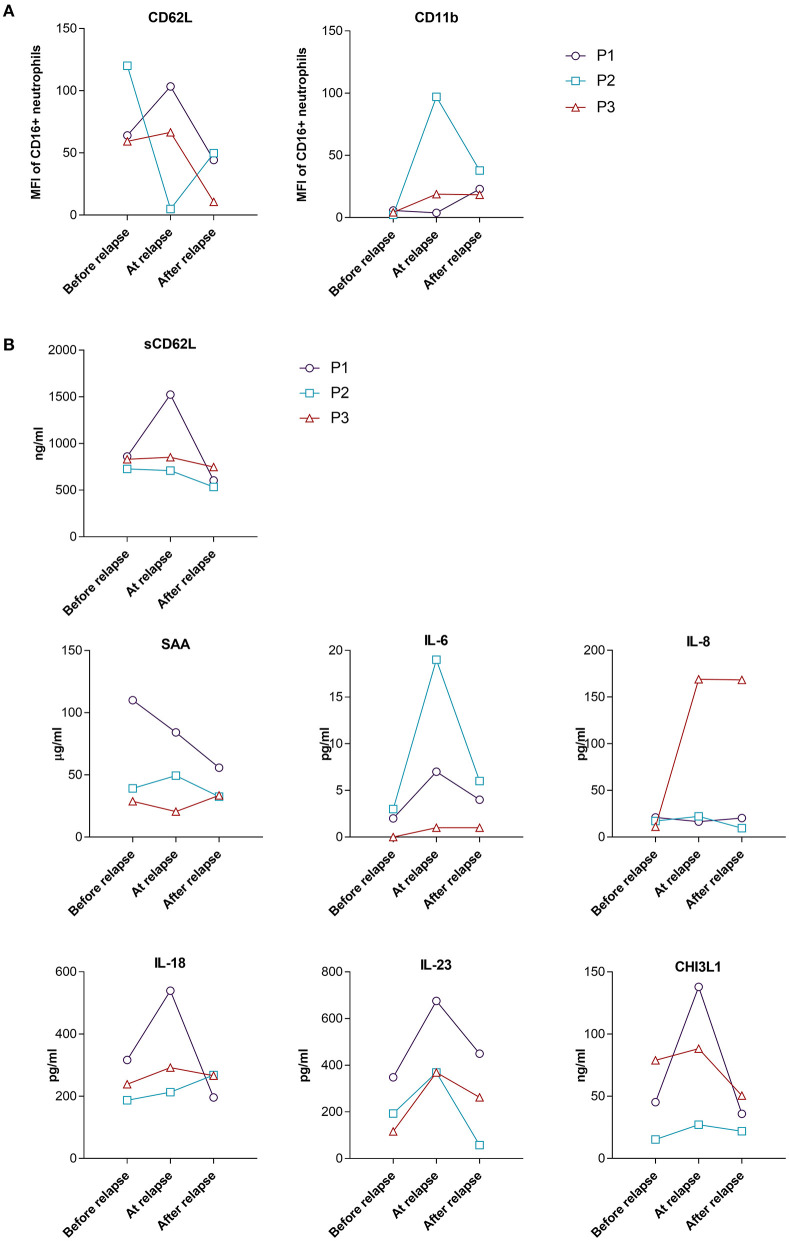
Surface expression of CD62L and CD11b on neutrophils **(A)** and serum levels of selected analytes **(B)** from relapsing GCA patients during the 48 weeks of follow-up. Shown are levels/MFI at the last follow-up time point before relapse, at the time point closest to relapse and 12 weeks after relapse. P1 relapsed at week 24, P2 relapsed at week 48 and P3 relapsed at week 12. MFI, median fluorescence intensity, P, patient.

The expression of neutrophil CD62L and CD11b, as well as the levels of sCD62L in the group of relapsing patients varied greatly between the three patients (Figures 5A,B). This variation might also be attributed to the different time points when the patients relapsed and different GC dosages.

Acute phase reactants IL-6 and SAA showed different fluctuations in relapsing patients (Figure 5B). At the time of relapse (active disease) strong increase of IL-6 as compared to the last follow-up point before relapse (inactive disease) was observed for P1 and P2, while the increase in P3 who relapsed early in the course of the disease (week 12), while still on a high GC dose (48 mg/day) was very low. A small increase in the level of SAA was observed in just one patient at the time of relapse, compared to the time point before relapse (Figure 5B). Since elevated levels of IL-6 at the time of relapse could indicate the reactivation of GCA, we next determined how many patients in the responder group had elevated levels of IL-6 without disease flare during the follow-up period. We defined the number of patients with elevated levels of IL-6 on two consecutive visits at weeks 12, 24 or 48 [similar to Stone et al. (17)] compared to the week 4 when patients were on high GC dose (48 mg/day) for the longest period of time (4 weeks). 15/25 (60%) patients from the responder group (10/10 from GC only group and 6/15 from GC plus leflunomide group) exhibited elevated levels of IL-6 (at two consecutive visits at weeks 12, 24 or 48 vs. week 4), despite disease inactivity and no subsequent relapse. We therefore next looked for other biomarkers that were elevated in all three relapsing patients with active (at the time of relapse) compared to non-active (before relapse, in remission) disease. We identified three serum markers which met our criteria: IL-18, IL-23 and CHI3L1 (Figure 5B). Subsequently, we analyzed how many patients from the responder group had elevated levels of the three identified parameters during the follow-up without a subsequent relapse (the same as previously described for IL-6). 13 out of 25 (52%) patients in the responder group had elevated IL-18, 9/25 (36%) patients had elevated CHI3L1 and 6/25 (24%) had elevated IL-23 during the course of the disease in the absence of clinical manifestations indicating a relapse.

## Discussion

The current longitudinal study provides data on the effects of short- and long-term use of GC or GC, in combination with leflunomide on leukocyte subtype dynamics, neutrophil phenotype and serum analytes in GCA patients. The steroid-sparing effect of leflunomide in GCA has been shown in an open-label study by Hočevar et al. (14). During the first 48 weeks of follow-up, 13.3% of GCA patients who received GC in combination with leflunomide relapsed compared to 39.1% relapsing patients receiving GC monotherapy (14). We observed similar findings in the present study, where all three relapsing patients were receiving GC monotherapy and there were no relapses observed in patients under combinatorial therapy with leflunomide during the 48 weeks of follow-up. Furthermore, the relapsing patients received leflunomide at the time of relapse in addition to GC and they all entered into remission 12 weeks after relapse. Subsequently, they were able to adhere to GC tapering as initially scheduled.

We observed an immediate short-term effect of high dose GC on increasing the numbers of CD4^+^ T- and B-lymphocytes after 1 week, while long-term GC treatment (12 weeks) resulted in decreased numbers of CD4+ T- and B-lymphocytes, with increased numbers of neutrophils compared to the pre-treatment time. Similar findings were reported previously by other studies (19, 27). Van der Geest et al. observed the same increase in B cell count 2 weeks after GC treatment, however did not find any evidence of either B cell replenishment from the bone marrow or compensatory hyperproliferation of circulating B cells (27). An increase in B cell counts as a result of early GC treatment might reflect a redistribution or intravascular marginalization of B cells during active disease. Neutrophilia caused by GC is a known effect resulting from increased polymorphonuclear cell release from the bone marrow to the circulation and their increased survival. On the other hand, GC can reduce the migration of neutrophils into inflammatory sites by decreasing the surface expression of CD62L, thus preventing the adhesion of neutrophils and their tissue accumulation (28). The same was observed in the present study, in which the expression of CD62L rapidly decreased after initiation of GC treatment and began to increase progressively to week 48, when patients received lower doses of GC monotherapy. The expression of CD62L was higher at week 48 compared to the pre-treatment timepoint. The activated CD62L^hi^CD11b^hi^ neutrophil profile in GCA patients at baseline has been reported in a previous study (20). Within 1 week of GC treatment, this phenotype was brought under control, as demonstrated by switching to CD62L^lo^CD11b^lo^ phenotype with reduced endothelial adhesion. However, 24 weeks after initiation of GC therapy and tapering, an escaped pro-inflammatory phenotype (CD62L^hi^CD11b^hi^), with elevated endothelial adhesion was reported (20). The progressive increase of CD62L was not observed in our study in patients under combinatorial therapy with leflunomide. In contrast, the expression of CD62L decreased in these patients compared to the pre-treatment time. Leflunomide is a selective inhibitor of *de novo* pyrimidine synthesis (limiting proliferation of lymphocytes) and lowers the production of IL-6, TNF-α, IL-12 and IL-17. Moreover, leflunomide can affect the expression of adhesion molecules and reduces leukocyte adhesion to endothelial cells (29), which could explain the downregulation of CD62L neutrophil expression observed in our study. The addition of leflunomide might have a beneficial long-term effect on the control of neutrophil adhesion. However, no significant differences in the expression of CD62L were observed between relapsing patients and responders.

We and others have previously already determined higher serum levels of CHI3L1 in therapy-naïve GCA patients compared to HBDs (22, 30), while in the present study, we additionally found that the levels of CHI3L1 were associated with signs of transmural inflammation and vessel occlusion in temporal arteries. The source of CHI3L1 in serum of GCA patients might stem from monocytes, macrophages and giant cells (30). Additionally, CHI3L1 is highly expressed in TABs of GCA patients, predominantly in the intima-media border region (30, 31). CHI3L1 is involved in tissue remodeling and angiogenesis (32), and deregulation of these processes in GCA TABs might contribute to increased amounts of CHI3L1 in occluded temporal arteries. Our finding suggests that CHI3L1 is mainly released in fully developed GCA with transmural inflammation and lumen occlusion. Although *in vitro* production of CHI3L1 by macrophages has been shown to be sensitive to GC (33), we observed only a slight reduction in serum CHI3L1 levels between baseline and follow-up visits, indicating that cells producing CHI3L1 may be GC resistant. In line with these observations, GCA patients with extensive transmural inflammation and remodeling of temporal arteries had higher levels of CHI3L1 that might require a therapeutic approach different from the currently established GC. Targeting CHI3L1 in GCA may inhibit macrophages that might currently be insufficiently suppressed by GC (34).

Traditional inflammatory parameters, such as ESR and CRP have been described as insufficient markers for monitoring disease activity in GCA (17). In the current study, increased ESR and CRP were observed in only two out of three relapsing patients at the time of relapse when the disease clinically reactivated. Tocilizumab additionally suppresses these markers (17, 35), indicating the need for new inflammatory markers to aid in monitoring GCA activity during treatment. Our results demonstrated the suppressive effect of GC on systemic levels of additional acute phase reactants, such as SAA and IL-6 which limits their use as markers of disease activity during therapy. Similar to our results, Dartevel et al. showed significantly higher SAA levels in patients with active (newly diagnosed and relapsing) compared to inactive GCA (responding to therapy). However, the authors did not compare the difference in SAA levels in relapsing patients at the time before, at and after relapse (24). In contrast to SAA, levels of IL-6 increased in all three relapsing patients, in our study, when the disease reactivated, but also in 60% of the responder patients at two consecutive visits at weeks 12, 24 or 48 compared to week 4. All 10 patients in the responder group that received GC monotherapy had increased levels of IL-6, while this was observed in only 5/15 patients under combinatorial therapy with leflunomide. Since increasing the doses of GC to completely suppress serum IL-6 would lead to the higher rate of treatment related adverse effects, the addition of leflunomide could be a better option. As seen in the present study, leflunomide may have a beneficial effect on reducing both systemic and vascular inflammation. CHI3L1, on the other hand, was increased in all three relapsing patients at the time of relapse and was elevated in only 36% of the patients who responded to therapy with GC or GC and leflunomide. This might indicate an incompletely controlled disease process in these patients that may lead to a future relapse, but needs to be confirmed on a larger cohort of patients after a longer follow-up period.

Prior to treatment, relapsing patients had significantly elevated levels of IL-23 compared to patients without relapses. The levels of IL23 were also higher in all three relapsing patients, at the time closest to relapse, compared to the last time point before relapse. Levels of IL-23 decreased again after patients entered into remission and concurrently received leflunomide. Conway et al. similarly found significantly increased expression of IL-23 in the TABs of GCA patients with two or more relapses compared to patients without or with only one relapse (36). IL-23 is pivotal in differentiation of Th17 cells, producing IL-17A with pleiotropic effects on a variety of cells, including macrophages, neutrophils, endothelial cells and fibroblasts, and actively contributes to inflammatory cascades (37). IL-23 seems to be GC-dependent, since in our study, its levels decreased substantially from baseline visit to week 48 of follow-up in patients treated with GC monotherapy, however it remained elevated in GCA patients who experienced a relapse. Higher levels of IL-23 might indicate an ongoing vascular tissue inflammation in relapsing patients, inferring that IL-23 might serve as a marker of persistent, active disease and as a relapse predictor in GCA patients. Although patients receiving GC and leflunomide had slightly higher levels of IL-23 during the 48 weeks follow-up period, none of them developed a relapse. This might be associated with the primary effect of leflunomide inhibiting T-lymphocyte proliferation (29, 38) and thus inhibiting the IL-23-driven polarization toward the Th17 lineage (37, 39). As IL23 is most strongly expressed and produced by macrophages and dendritic cells (40, 41), this (in addition to CHI3L1) might represent another clue for potential therapeutic benefits of suppressing macrophage activation in GCA.

The strengths of our current report are the prospective study design, and the uniform clinical evaluation, with known dates of GC therapy start and tapering at follow-ups. Moreover, GCA patients joined our study when they were therapy-naïve, which allowed us to evaluate the effects of active disease. Previous longitudinal studies often included patients already treated with GC.

The major limitation of our study is a relatively small number of included and longitudinally followed GCA patients (*n* = 25), as well as the small number of relapsing patients (*n* = 3) that explains the lack of statistical significance. The patients were only followed up to 48 weeks when they did not yet achieve GC-free remission and we only assessed their peripheral blood that may not completely reflect the pathological processes at the sites of tissue inflammation. Future longitudinal studies with similar designs could provide further insights by increasing the number of patients, tested parameters and follow-up time, as well as assessing the vascular pathological processes in the temporal arteries in greater detail.

## Data Availability Statement

The raw data supporting the conclusions of this article will be made available by the authors, without undue reservation.

## Ethics Statement

The studies involving human participants were reviewed and approved by National Medical Ethics Committee of Slovenia. The patients/participants provided their written informed consent to participate in this study.

## Author Contributions

TK, KL, SS-Š, and AH designed the experiments. TK, KL, and PŽ performed the flow cytometry analysis and conducted the serum biomarker experiments. AK supported the analysis of flow cytometry study. TK and GT performed the statistical and bioinformatic analyses. AH and MT conducted the clinical evaluation of the patients. SS-Š, SČ, AH, and MT coordinated the study. MF-B provided critical input to data analysis, visualization and interpretation. TK wrote the original draft. All authors reviewed, edited the final draft, authors have seen, approve the manuscript and its contents, and as well as are aware of the responsibilities connected with the authorship.

## Funding

This work was supported by the Slovenian Research Agency ARRS with funding grant #P3-0314.

## Conflict of Interest

MF-B is employed by the company BioMed X Institute, Heidelberg, Germany. The remaining authors declare that the research was conducted in the absence of any commercial or financial relationships that could be construed as a potential conflict of interest.

## Publisher's Note

All claims expressed in this article are solely those of the authors and do not necessarily represent those of their affiliated organizations, or those of the publisher, the editors and the reviewers. Any product that may be evaluated in this article, or claim that may be made by its manufacturer, is not guaranteed or endorsed by the publisher.
