# Supplementary material for: From Active to Non-active Giant Cell Arteritis: Longitudinal Monitoring of Patients on Glucocorticoid Therapy in Combination With Leflunomide
Source: Front Med (Lausanne). 2022 Jan 20;8:827095. doi: 10.3389/fmed.2021.827095 (PMC8811148; doi:10.3389/fmed.2021.827095)
Supplement: Supplementary file 1 [file Table_1.DOCX]

**Supplementary Table S1: Differences between GCA patients receiving GC monotherapy and GC in combination with leflunomide at baseline visit (T_0_) and at week 12 of follow-up.**

|  | **Baseline visit (T_0_)** | | | **Week 12** | | |
| --- | --- | --- | --- | --- | --- | --- |
| **Measured parameters; median**  **(Q_25_-Q_75_)** | **GC**  (n=10) | **GC+leflunomide**  (n=15) | **P value** | **GC**  (n=10) | **GC+leflunomide**  (n=15) | **P value** |
| Neutrophil CD62L (MFI) | 97.2  (65.9-126.1) | 79.4  (45.3-89.4) | 0.0623 | 58.1  (33.2- 90.3) | 49.4  (26.4-61.3) | 0.4366 |
| Neutrophil CD11b (MFI) | 16.9  (6.6- 43.1) | 13.7  (2.9-25.5) | 0.3383 | 10.3  (7.1-25.8) | 11.7  (5.2-32.3) | 0.5080 |
| sCD62L (ng/ml) | 699.8  (662.7-739.4) | 779.1  (636.9-955.0) | 0.4284 | 616.4  (595.0-704.5) | 724.8  (573.7-860.6) | 0.3669 |
| SAA  (µg/ml) | 578.5  (106.5-1043) | 218  (29.7-346) | 0.1025 | 36.9  (16.9-90.6) | 37.0  (20.0-47.5) | 0.8065 |
| IL-6  (pg/ml) | 25.0  (11.5-57.3) | 14.0  (9.0-22.0) | 0.1248 | 2.5  (2.0-10.0) | 5.0  (2.0-10.0) | 0.0787 |
| IL-8  (pg/ml) | 54.5  (21.9-100.2) | 21.3  (15.9-192.5) | 0.4611 | 21.6  (13.1-40.1) | 19.1  (13.9-32.3) | 0.9783 |
| IL-18  (pg/ml) | 196.4  (141.3-236.9) | 175.4  (75.6-206.9) | 0.2435 | 201.7  (142.6-227.7) | 267.3  (119.5-317.5) | 0.0805 |
| IL-23  (pg/ml) | 57.8  (21.6-223.8) | 116.6  (71.3-192.8) | 0.4043 | 81.9  (21.6-194.5) | 71.3  (57.8-216.4) | 0.8589 |
| CHI3L1 (ng/ml) | 110.7  (30.0-189.4) | 71.9  (38.8-160.4) | 0.8918 | 69.1  (26.4-92.9) | 70.3  (41.1-158.0) | 0.5671 |
