# Supplementary figures and images for: From Active to Non-active Giant Cell Arteritis: Longitudinal Monitoring of Patients on Glucocorticoid Therapy in Combination With Leflunomide
Source: Front Med (Lausanne). 2022 Jan 20;8:827095. doi: 10.3389/fmed.2021.827095 (PMC8811148; doi:10.3389/fmed.2021.827095)

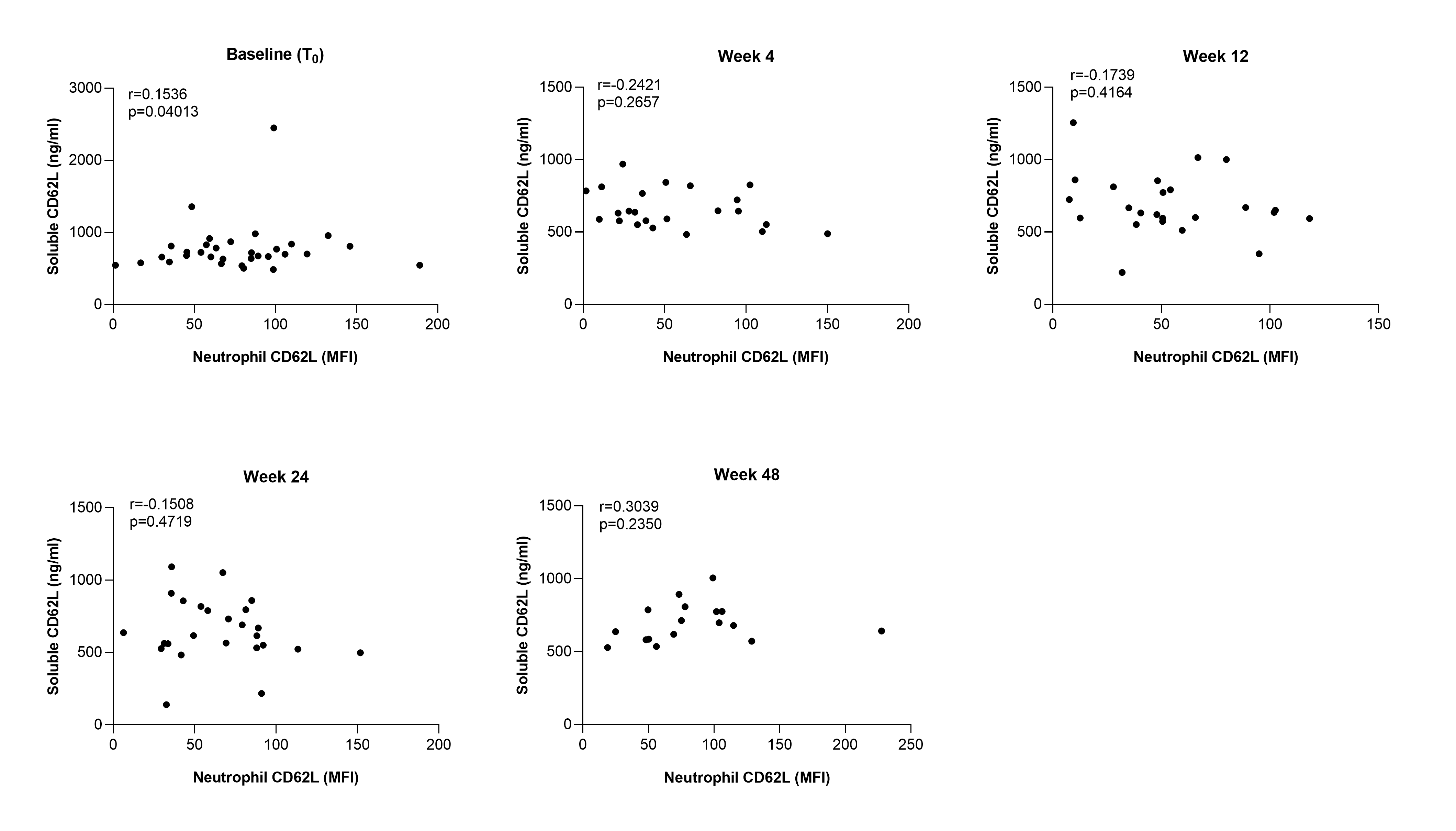

Supplement: Supplementary file 2 [file Image_1.TIF]
